# Physicians’ knowledge and sepsis guide implementation in tertiary care hospitals in China

**DOI:** 10.1186/s12909-022-03472-x

**Published:** 2022-05-20

**Authors:** Silu Han, Lijian Cui, Yifan Qu, Tian Tian, Bing Wei, Junyu Wang, Jun Yang

**Affiliations:** Beijing Chao-Yang Hospital, 5 Jingyuan Road, Shijingshan District, Beijing, 100043 China

**Keywords:** Sepsis, Sepsis guidelines, Heterogeneous, Standardized training

## Abstract

**Background:**

This study was designed to investigate the extent to which physicians involved in sepsis management understand and adopt sepsis guidelines in clinical practice. The overarching aim of this study was to generate ideas for developing more effective training methods to help physicians apply the guidelines in patient management.

**Methods:**

Physicians working in a tertiary care hospital, primarily in the emergency and critical care departments, were recruited into the survey. They were asked to fill questionnaires which were designed to collect sepsis score, diagnostic indicators, fluid resuscitation, antibiotics choice, access to knowledge and training, as well as implementation of sepsis guidelines in clinical diagnosis and treatment.

**Results:**

Overall, the response rate was 625/661 (94.5%). The investigate shows the basic information of all physicians who participated in the answer sheet, including their work department, professional title and whether their hospital was a teaching hospital. Significant differences were identified among the physicians in terms of method of acquiring sepsis guidelines, the impact of study guidelines on clinical diagnosis and treatment, efficiency of training methods, cognition of fluid resuscitation in patients with sepsis, the cognition of sepsis rehydration principles, selection of antibiotics for patients with sepsis, the basis for antibiotic selection, among other variables.

**Conclusion:**

Although majority of physicians involved in tertiary care hospital understand the contents of sepsis-3 guidelines, the clinical implementation of the guidelines in the diagnosis and treatment of patients with sepsis is highly heterogeneous. Thus, there is need to develop standardized training for physicians involved in sepsis diagnosis and treatment.

**Supplementary Information:**

The online version contains supplementary material available at 10.1186/s12909-022-03472-x.

## Background

The 2016 joint release by the SCCM (American Society for Critical Care Medicine) and ESICM (European Society for Critical Care Medicine) on the definition and diagnostic criteria for sepsis-3 defines sepsis as life-threatening organ dysfunction due to dysregulated response to infection. Clinically, organ dysfunction can be indicated by an increase in the SOFA (sequential [sepsis-related] organ failure assessment) score of ≥ 2 [1]. Prevention, early detection, and effective treatment of sepsis are key to improving patient survival [2]. The guidelines emphasized that for better prognosis, sepsis diagnosis and treatment should be standardized. However, the implementation of sepsis-3 faces various challenges. In our clinical work, we found differences in the understanding of sepsis-3 guidelines and the resulting treatment options among different physicians, the most obvious differences being reflected in sepsis fluid resuscitation and the choice of antibiotics. By searching the existing literature,a study on the identification of sepsis by nurses in emergency department in Denmark found that initially, only 18% knew the standard for sepsis, and 80% did not know the blood pressure level during septic shock. After a 12-week follow-up, 75% of the people knew the diagnostic criteria for sepsis, and their awareness of blood pressure level in patients in septic shock increased to 100% [3]. However, that study involved only the identification of sepsis by nursing staff in emergency departments abroad. Similar Chinese studies are lacking, and it has not been reported abroad. Here, we investigated the factors underlying the poor homogeneity in sepsis diagnosis and treatment, and recommended guidelines for evidence-based standardization of clinical sepsis diagnosis and treatment.

## Materials and methods

### Participants

The study involved physicians from 126 Chinese tertiary care hospitals in 26 provinces and municipalities (except Xin jiang, Tibet, Taiwan.) and majority worked in emergency (88.32%) and critical care (10.88%) departments.

### Methods

We used a questionnaire to investigate the understanding and degree of implementation of sepsis guidelines by physicians in China.All respondents were physicians from tertiary care hospitals, mainly working in emergency(emergency center) and critical care centers (intensive care unit). The contents and evaluation criteria of the questionnaire were developed by infection experts. The questionnaire asked for responses on the following: i) basic information, (ii)method of acquiring sepsis guidelines, (iii) efficiency of training methods, (iv) impact of study guidelines on clinical diagnosis and treatment,(v) cognition of fluid resuscitation in patients with sepsis,(vi) cognition of sepsis rehydration principles, (vii) selection of antibiotics for patients with sepsis, especially the use of restricted antibiotics(compared with non-restricted antibiotics, restricted antibiotics have certain limitations in terms of efficacy, safety, and bacterial resistance, and their use should be restricted, such as third-generation cephalosporins)and (viii) basis for antibiotic selection. The questionnaire was based on the star platform (is a professional online questionnaire survey, examination, evaluation and voting platform, website: https://www.wjx.cn/)and was sent from the platform to the education departments of participating hospitals. The education department of participating hospitals then coordinated the completion of questionnaires by full-time emergency department and intensive care physicians, after which data were retrieved by the questionnaire star platform [4]. The entire investigation was double-blinded [5]. This study was approved by Beijing Chao-Yang Hospital ethics committee and all participants consented to the study.

### Effect evaluation

All respondents completed the questionnaire (as single item responses or multiple choices) and reached different conclusions on the study of sepsis guidelines and clinical practice.

## Statistical methods

Statistical analyses were done on GraphPad prism (version 5.0). All data are expressed as percentage.

## Results

We identified the reasons for the poor homogeneity in sepsis diagnosis and treatment and recommended guidelines for evidence-based standardization of clinical sepsis diagnosis and treatment. The response rate was 625/661 (94.5%). Of the participating physicians, 88.32% worked in the emergency department and 10.88% in the intensive care department. Among them, 32% had junior(Work less than five years) and 37.12% had intermediate professional titles,(Work time is between 5–10 years) 30.88% had at least deputy senior professional titles(Work more than 10 years). 95.36% were teaching hospitals and 4.64% were non-teaching hospitals (Table 1).

**Table 1:** Basic information on interviewees

Survey content (percentage, n%)	
The doctor’s level	
Senior title	30.88%
Intermediate title	37.12%
Primary title	32%
Department	
Emergency Department	88.32%
Intensive Care	10.88%
Other Department	0.8%
Teaching Hospital	
Yes	95.36%
No	4.64%

**Fig. 1:**
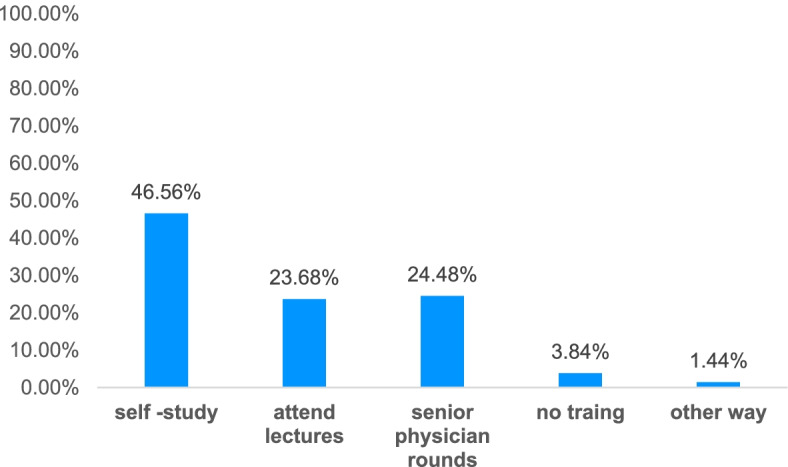
Knowledge acquisition

In terms of how to acquire knowledge on sepsis guidelines, majority of the physicians received sepsis-related training. 46.56% physicians had reported acquiring sepsis knowledge by self reading, 23.68% by attending lectures, 24.48% by learnt from senior physicians,and 1.44% by studied in other ways. Only 3.84% did not receive training (Fig. 1).

**Fig. 2:**
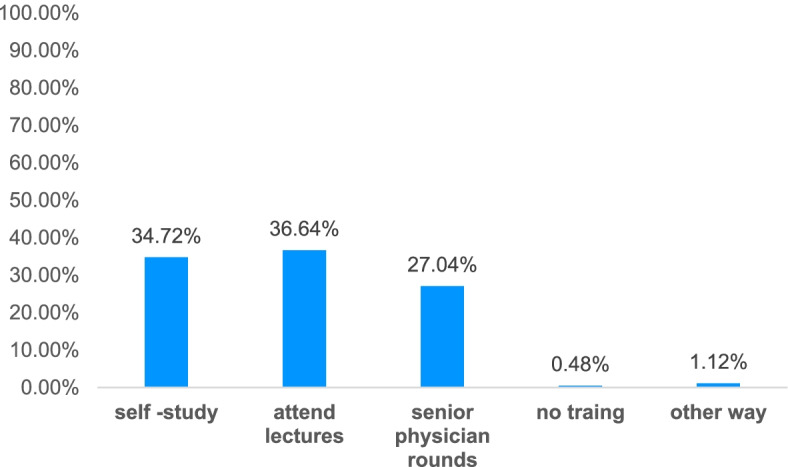
Impact on diagnosis and treatment

For the effect of sepsis guidelines on clinical diagnosis and treatment, 36.64% of physicians felt that attending lectures was effective, 34.72% felt self-study was effective, 27.04% felt ward rounds with senior physicians was effective (Fig. 2).

**Fig. 3:**
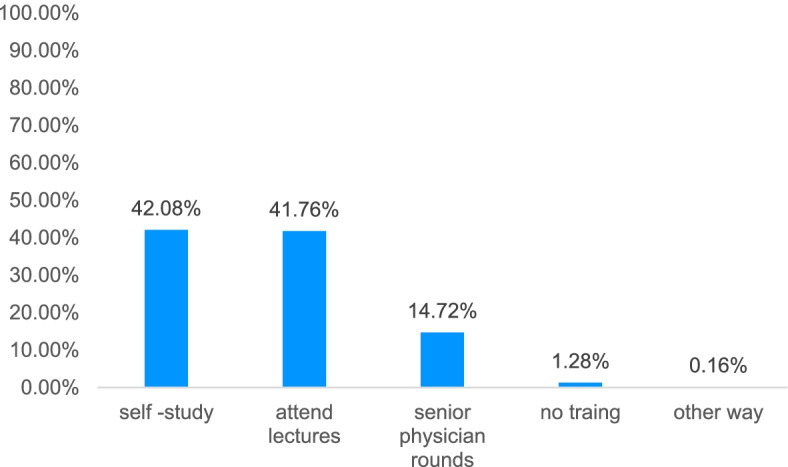
Efficiency of training methods

In terms of efficiency of training methods, the physicians surveyed thought that reading the guidelines and attending lectures was best for learning (42.08% vs 41.76%), followed by senior physicians’ ward rounds (14.72%, Fig. 3).

**Fig. 4:**
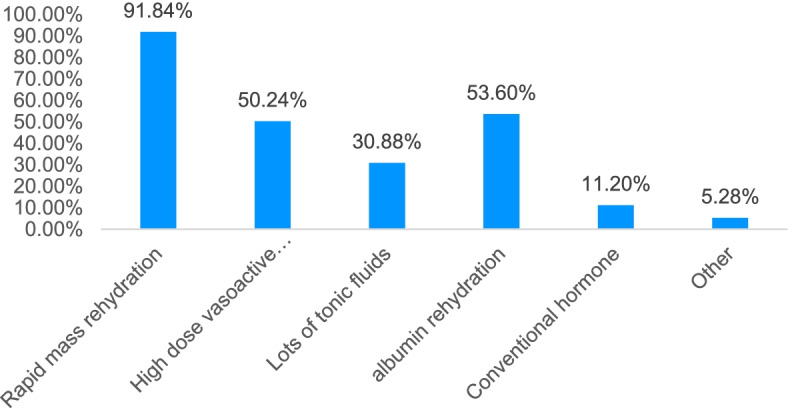
Cognition of fluid resuscitation in patients with sepsis

**Fig. 5:**
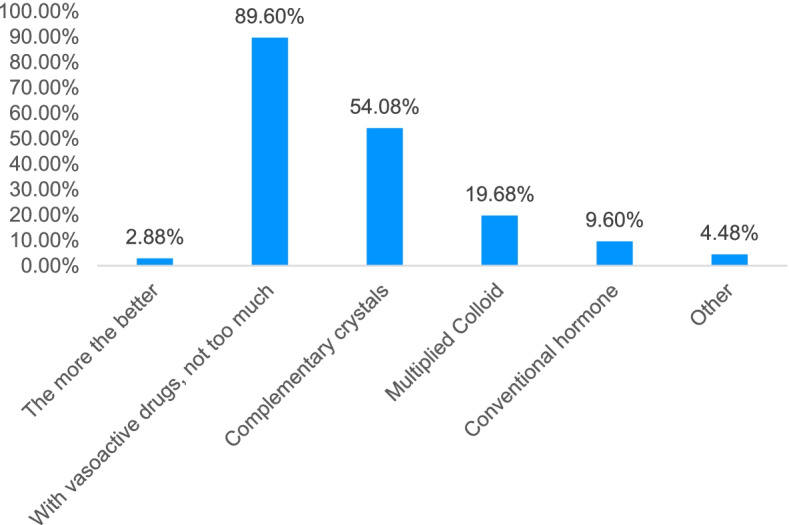
Sepsis rehydration principle cognition

With regards to single treatment strategy, > 90% of the surveyed physicians thought that graph learning was more effective. Treating septic shock patients first requires rapid and massive fluid replacement, followed by high doses of vasoactive drugs and human serum albumin supplementation. However, most physicians are not satisfied and consider that fluid replacement in sepsis should follow the principle of “use with vasoactive drugs, not too much” (Figs. 4–5).

**Fig. 6:**
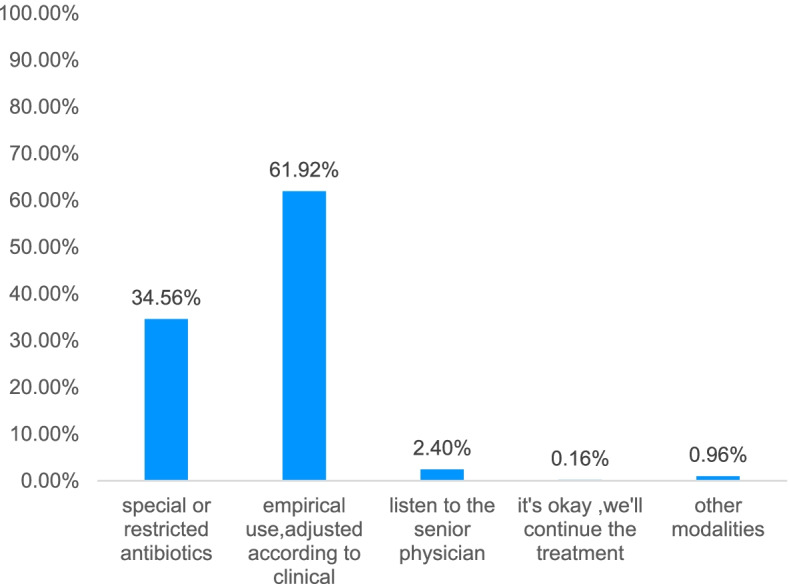
Antibiotics selection

**Fig. 7:**
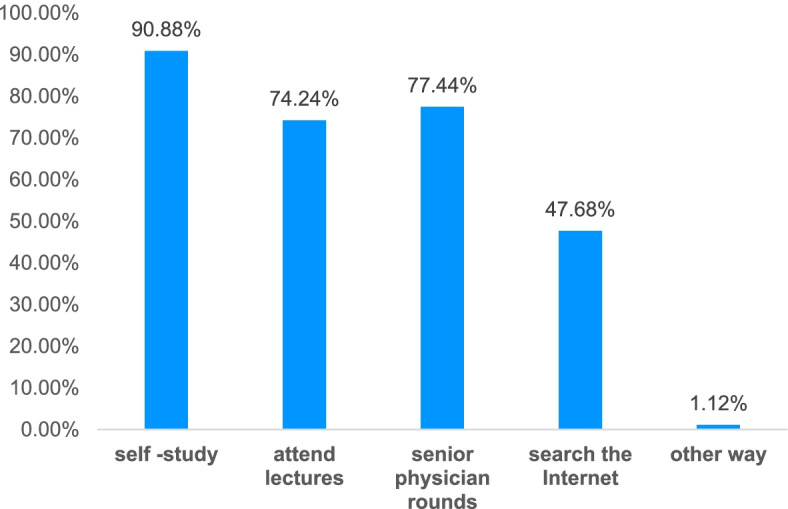
Basis of antibiotic use selection

More than half of the physicians(61.92%) chose to use antibiotics empirically, which was adjusted based on the clinical situation. 34.56% of the physicians thought that special or restricted antibiotics should be used immediately in patients with sepsis. 2.40% of the medical students chose to listen to senior physicians, and 0.16% did not choose (Fig. 6). Further investigation on the basis of antibiotic use found that self-study guide, attending lectures and studying, and senior physicians’ ward rounds were the most important factors (> 70%). It should be noted that 47.68% of physicians relied on internet searches for drug use guidance (Fig. 7).

## Discussion

Although sepsis guidelines have been published for a long time, challenges remain in their clinical application. This survey shows that although physicians at Chinese tertiary care hospitals think they possess “good” practical skills in the study and implementation of sepsis guidelines, they are actually “poor” in these areas. This is probably because most trainees only pay attention to concepts without practicing when learning sepsis guidelines. Thus, because learning is not combined with clinical practice, or because the training period is lengthy, memory of the guidelines may become vague.

Findings from this study indicate that room for improvement exists regarding knowledge of sepsis among physicians in tertiary care hospitals. Our findings show that attending lectures, self-study guides, and learning from senior physicians are considered the most effective learning methods and can quickly influence clinical diagnosis and treatment. However, nearly half of the physicians choose self-study guides for acquiring knowledge on sepsis and their proportion is significantly higher than that of who use other methods. However, clinicians are more willing to attend lectures and recognize the effectiveness of this learning method. This is because medicine is an empirical subject based on theoretical knowledge and most of the instructors are physicians with extensive clinical experience. Hence, despite some conflicts in guidelines, treatment methods in line with Chinese population characteristics (height, weight, past medical history, allergies.)are easier to follow for Chinese physicians. It should be noted that the impact of senior physicians’ ward round teaching [6], especially to junior physicians, is significantly lower than we expected, indicating that compared to attending lectures, guidance from senior physicians’ is not enough, or does not implore participants to think and discuss problems. This has negative impact on clinical diagnosis and treatment.

Fluid resuscitation is an important part of sepsis management [7]. This survey revealed that > 90% of physicians believed that rapid and large-scale fluid replacement is needed, followed by administration of high-dose vasoactive drugs and human serum albumin supplements. However, when talking about the principle of fluid replacement in sepsis, most physicians thought that it should be used with vasoactive drugs, and that the amount of fluid replacement should not be too high. Such contradictory conclusions show that there is a variability in our understanding of the guidelines on clinical diagnosis and treatment, which is also obviously reflected in the use of antibiotics. Our study shows that more than half of the participating physicians chose to first use antibiotics empirically, followed by adjustment depending on clinical outcome. Nearly 1/3 of participating physicians chose the empirical use of antibiotics. With regards to drug use plan, most physicians feel that their choice of antibiotic treatment plan is based on studying guidelines. The cause of confusion in the diagnosis and treatment process may stem from a lack of standardized training, which leads to unclear concepts and biased interpretation of treatment strategies, which needs urgent correction [8, 9].

In summary, we have identified a series of problems in the study and clinical implementation of sepsis guidelines. The study of sepsis guidelines requires standardized training. This can be done through investigation and analysis, we found that although the proportion of physicians practicing self-study is higher, they are actually more willing to attend lectures and training. Therefore, we must first increase the number of senior physicians’ rounds from once a week to twice a week. When teaching ward rounds senior physicians should explain guidelines during clinical practice, analyze patient condition in clinical settings, interpret guidelines in detail, and ensure the safety of each participant during diagnosis and treatment with the participating physicians. This is more conducive for junior physicians to gain clinical experience and offers better “closed-loop learning” [10]. Additionally, the quality and effectiveness of training should be enhanced using seminars, case sharing, online exchange learning, and sepsis-related knowledge competitions.

Additionally, in order to prevent the learning of guidelines from being mere formality, training may be improved by optimizing content (e.g., using charts, tables, and other intuitive teaching methods), and minimizing text content in order to facilitate memory. A foreign survey of emergency department nurses found that training employees using pocket cards, posters, and electronically accessible guides improves their recognition of sepsis [3].

In standardized training the duration of training can be reduced by having three training sessions per month for junior physicians, two training sessions per month for intermediate physicians, and one training session per month for senior physicians, which may improve memory of sepsis guidelines [11].

With regards to training effect, attention should be paid on follow-up, clinical implementation of training guidelines, and full comparison of prognosis and symptom improvement times in patients treated based on clinical experience and standardized guidelines [12]. Onsite training (i.e., education in actual clinical settings) can enhance learning. With the popularity of simulation centers and computerized laboratories, core technologies have increasingly important roles in teaching clinical skills [13], such as setting up virtual online patients, and allowing students to give diagnosis and treatment plans according to described cases. This method can help educators identify knowledge gaps among students of sepsis management and focus on weak links. The curriculum should be strengthened to cultivate students’ ability to identify and treat sepsis, thereby improving safety and treatment effectiveness [14]. Currently, online learning is especially suitable and critical in the fight against the COVID-19 (Coronavirus disease 2019) pandemic [15].

## Limitation

The purpose of this survey is to find out the current problems of physicians’ knowledge and sepsis guide implementation in tertiary care hospitals in China, analyze what adverse effects may be caused, and how to improve this situation. The limitation of our manuscript is that we have not verified whether the proposed method is really effective, which is also the direction of our next work.

## Conclusion

This survey undertaken in China found that, despite positive attitudes toward sepsis, opportunities exist for improving knowledge level and clinical practices among physicians in Chinese tertiary care hospitals. Stemming from the barriers perceived from their own perspectives, establishing standardized protocols, and systematically initiating and implementing training on sepsis, may markedly improve sepsis management in China.

## Supplementary Information

**Additional file 1.**

## Data Availability

All data and material underlying this study are included in this paper.

## Abbreviations

SCCM: American Society for Critical Care Medicine
ESICM: European Society for Critical Care Medicine
SOFA: Sequential [Sepsis-related] Organ Failure Assessment
COVID-19: Corona Virus Disease 2019
