# Supplementary material for: Physicians’ knowledge and sepsis guide implementation in tertiary care hospitals in China
Source: BMC Med Educ. 2022 May 20;22:388. doi: 10.1186/s12909-022-03472-x (PMC9122554; doi:10.1186/s12909-022-03472-x)
Supplement: Supplementary file 1 — Additional file 1. [file 12909_2022_3472_MOESM1_ESM.pdf]

首都医科大学附属北京朝阳医院伦理委员会
科研课题申报快速审查批件（3.0版）

受理号： 2016-7-4-1　　　　编号： 2016-科-143

科研项目名称	新脓毒症诊断标准对于筛选早期目标导向治疗人群及预后的意义		
申报课题类型	北京市属医院科研培育计划		
我院项目负责人	王军宇		
项目负责人所在科室	急诊科		
报送材料	科研项目伦理审查申请表		
	北京市属医院科研培育计划项目申报书		
	知情同意书		
主审委员	钟光珍		
主审结果	同意（1）条	不同意（0）条	作必要修改后同意（0）条
	作必要修改后重审（0）条	终止或暂停试验（0）条	
审查决定	■同意 □作必要修改后同意 □作必要修改后重审 □终止/暂停试验 □不同意		

审查意见：
无

注意事项：
1. 本申报审查批件将在伦理办公室备案。
2. 请在项目获得有关部门批准并在我院正式立项后重新递交伦理审查资料，经我院伦理委员会会议审查批准后方可开展研究。

主任委员/副主任委员签字：

伦理委员会（盖章）

2016 年 7 月 5 日
